# Noncoding RNA in drug resistant sarcoma

**DOI:** 10.18632/oncotarget.19029

**Published:** 2017-07-06

**Authors:** Xiaoyang Li, Jacson K. Shen, Francis J. Hornicek, Tao Xiao, Zhenfeng Duan

**Affiliations:** ^1^ Department of Orthopedics, The Second Xiangya Hospital, Central South University, Changsha, Hunan, 410011, China; ^2^ Sarcoma Biology Laboratory, Department of Orthopaedic Surgery, Massachusetts General Hospital and Harvard Medical School, Boston, Massachusetts, 02114, USA

**Keywords:** noncoding RNA (ncRNA), microRNA (miR), long noncoding RNA (lncRNA), drug resistance, sarcoma

## Abstract

Sarcomas are a group of malignant tumors that arise from mesenchymal origin. Despite significant development of multidisciplinary treatments for sarcoma, survival rates have reached a plateau. Chemotherapy has been extensively used for sarcoma treatment; however, the development of drug resistance is a major obstacle limiting the success of many anticancer agents. Sarcoma biology has traditionally focused on genomic and epigenomic deregulation of protein-coding genes to identify the therapeutic potential for reversing drug resistance. New and more creative approaches have found the involvement of noncoding RNAs, including microRNAs and long noncoding RNAs in drug resistant sarcoma. In this review, we discuss the current knowledge of noncoding RNAs characteristics and the regulated genes involved in drug resistant sarcoma, and focus on their therapeutic potential in the future.

## INTRODUCTION

Sarcomas are a group of heterogeneous malignant tumors that arise from mesenchymal origin. These malignancies are characterized by more than 50 subtypes with distinct clinical presentation and histology [1]. Although relatively rare, sarcomas account for nearly 21% of all pediatric solid cancers and are the third leading cause of cancer-related deaths in patients under the age of 20 [2, 3]. Despite the significant development of novel multidisciplinary treatments, including surgery, chemotherapy and radiation therapy, the 5-year survival rate has reached to a plateau in recent years [4]. This phenomenon is seen particularly in patients with metastatic or recurrent advanced disease, where the overall median survival is around 15 months, and only about 10% of these cases are still alive at five years [5].

Since the introduction of adjuvant and neoadjuvant chemotherapy in the 1970s, chemotherapy with multimodality therapeutic strategies have made considerable progress in improving the survival rate and quality of life for patients with certain types of sarcomas, especially for osteosarcoma, Ewing's sarcoma and rhabdomyosarcoma [6–12]. However, progress has remained stagnant over the last three decades with conventional chemotherapy approach [13, 14]. The development of drug resistance to chemotherapy is one of the major reasons for treatment failure, but the mechanisms of drug resistance have not been completely understood. Multiple subtypes make drug resistance mechanisms more diverse and controversial in sarcoma. To date, none of the ongoing attempts to address current mechanisms have been applied in the clinic with the aim of restoring drug efficacy in sarcoma. Therefore, identification of novel mechanisms of drug resistance in sarcoma that can be directly translated to clinic is required.

With the rapid development of next-generation sequencing technology to whole genomes and transcriptomes, it has been shown that only 2% of the human genome encodes mRNAs, whereas 98% of transcriptional products are noncoding RNAs (ncRNAs) [15]. ncRNAs have emerged as a class of cellular regulators involved in biological processes such as proliferation, differentiation, apoptosis and cell cycle, whereas mutations and dysregulation of ncRNAs have been linked to diverse human diseases, including sarcomas [16, 17]. Recent evidence has highlighted ncRNAs as being associated with drug resistance in sarcoma, mainly in two distinct subtype forms: microRNAs (miRs) and long noncoding RNAs (lncRNAs). miRs have moved to the forefront of ncRNA research in the past decade, while the role of lncRNAs is also emerging in different areas of cancer biology [18]. In this review, we summarize recent discoveries of the molecular functions of ncRNAs in cellular pathways related to drug resistance, and their potential as novel therapeutic strategies to overcome treatment failure of chemotherapies in sarcoma.

## NCRNAS AND DRUG RESISTANCE IN SARCOMA

ncRNAs are extensively transcribed within the human genome [19, 20]. They lack valid open reading frames and have no protein-coding capacity [21–23]; however, ncRNAs represent a broad class of structurally and functionally distinct RNAs (Table 1). These include long and short ncRNAs that represent around 60% of the RNAs distributed throughout the human genome [24, 25]. Short ncRNAs are no more than 200 nucleotides (nt) [26]. This group is composed of miR, PIWI interacting RNA, small nucleolar RNA, and others [26]. In contrast, transcripts longer than 200 nt are regarded as lncRNAs [27]. This group is diverse and highly abundant [28], and contains long intergenic ncRNAs, long intronic ncRNAs, pseudogene RNAs, to name a few [26]. Novel classes of ncRNAs are constantly being discovered, including circular RNAs [29] and enhancer RNAs [30].

**Table 1:** Classes of discovered human ncRNAs

Noncoding RNAs	Class	Symbol	Length (nt)	Functions	Significance
House-keeping ncRNAs	Transfer RNAs	tRNAs	73–94	Connect amino acids with mRNA	Translation
	Ribosomal RNAs	rRNAs	121–5070	Component of ribosome	Translation
	Vault RNAs	vRNAs	86–141	Component of vault	Expulsion of xenobiotics, such as chemotherapeutic compounds
Small ncRNAs	MicroRNAs	miRNAs	18–25	Modulate protein-coding genes, guide suppression of translation, Drosha and Dicer dependent small ncRNAs	Regulation of proliferation,differentiation, and apoptosis involved in human development
	Piwi-interacting RNAs	piRNAs	26–30	Bind to Piwi proteins, principally restricted to the germline	Involved in germ cell development, stem self-renewal, and retrotransposon silencing
	Small nuclear RNAs	snRNAs	150∼	Assemble with proteins into spliceosomes to remove introns during mRNA processing	Aid in the regulation of transcription factors or RNA polymerase II, maintaining the telomeres
	Small nucleolar RNAs	snoRNAs	60–200	Guide modifications of other noncoding RNAs, function as miRNA to regulate mRNAs, alternative splicing	Associated with the development of some cancers
	Promoter-associated small RNAs	paRNAs	20–200	Involved in the regulation of the transcription of protein-coding genes by targeting epigenetic silencing complexes	Relation to diseases has not yet been discovered
	Transcription initiation RNAs	tiRNAs	∼18	Involved in the regulation of the transcription of protein-coding genes by targeting epigenetic silencing complexes	Relation to diseases has not yet been discovered
Long ncRNAs	Long intergenic noncoding RNAs	lincRNAs	> 200	Involved in diverse biological processes, such as mRNA splicing and miRNA silencing	Involved in tumorigenesis and cancer metastasis
	Long intronic noncoding RNAs		> 200	Likely to be involved in post-transcriptional gene silencing	Aberrantly expressed in human cancers
	Telomere associated noncoding RNAs	TERRAs	> 200	Negative regulation of telomere length and activity through inhibition of telomerase	Possible impact on telomere-associated diseases, including many cancers
	Long noncoding RNAs with dual functions		> 200	Both protein-coding and functionally regulatory RNA capacity	Deregulation has been described in cancers
	Transcribed pseudogenes		> 200	Regulation of tumor suppressors and oncogenes by acting as microRNA decoys	Often deregulated during tumorigenesis and cancer progression
	Transcribed-ultraconserved regions	T-UCRs	> 200	Antisense inhibitors for protein-coding genes or other ncRNAs	Expression is often altered in some cancers, possible involvement in tumorigenesis
	Circular RNAs	circRNAs	> 100	Do not have 5′ or 3′ ends, act as miRNA sponge, transport miRNAs, regulate mRNA through limited base pairing	May be used to study pathogenesis and devise therapeutic interventions
	Enhancer RNAs	eRNAs	50–2000	Important components in enhancer activity, have effect on the trans-criptional regulation in cis and in trans	May provide promising regulatory routes in tumor suppression

ncRNAs were considered to be junk or debris derived from the DNA transcriptional process initially [25]. However, recent studies have revealed that ncRNAs could be deregulated or mutated in many human cancers and they play an important role in pathological processes, including in tumorigenesis, metastasis and treatment resistance [31–34]. The development of drug resistance in sarcoma is affected by many factors, most of which involve a change in expression of certain genes. Mutations, amplifications or deletions within oncogenes and/or tumor suppressors were considered to be associated with the development of drug resistance [35–37]. ncRNAs have emerged as major regulators of epigenetic, transcriptional, and post-transcriptional gene expression and alternative splicing, which provides cells with yet another mode to fine-tune their transcriptome and adjust their proteome in response to stimuli. ncRNAs, mainly in the form of miRs and lncRNAs, have been linked to sarcoma drug resistance through abnormal gene regulation related to the activity of drug efflux transporters; the activation of drug target mutations or changes; DNA repair and cell cycle arrest; apoptosis and cell-survival pathways; cancer stem cells (CSCs) and autophagy involved signaling pathways [38–42].

## ROLE OF MIRS IN DRUG RESISTANT SARCOMA

miRs are a large family of conserved endogenous single-stranded small ncRNAs derived from the human genome [43]. The initial discovery of miR was in 1993 [44], and to date, over 1,400 human miRs have been identified [45]. Over one-third of the genes in human genome are regulated by miRs [46]. miRs bind to the 3′-untranslated region (3′-UTR) of its mRNA target, based on a partial base-pairing complementarity [47]. Translation of the mRNA is ultimately prevented either by transcript degradation, inhibition of translation, or mRNA decay, leading to a reduced level of protein [48].

Mutation patterns or aberrant expressions of miRs have been detected in sarcoma and have been associated with clinical features such as progression, metastasis and drug resistance [49]. Upregulation of miRs induces variations in drug metabolism and disposition through the regulation of target mRNAs (Table 2). The products of these target genes can be drug transporters, nuclear receptors, transcription factors or drug-metabolizing enzymes [50]. This leads to altered expression of proteins that are involved in the signal transduction network of drug resistance, and ultimately changing the chemosensitivity of cancer cells [51].

**Table 2:** Summary of miRs involved in drug resistant sarcoma

miRs	Sarcomas	Alteration	Drugs	Resistance mechanisms	Major targets	References
miR-143	Liposarcoma Osteosarcoma	↓	Doxorubicin	Alterations in drug targets Cell cycle Evasion of apoptosis Cancer stem cells Autophagy	Top2A PRC1 PLK1 Bcl-2 ALDH1 CD133 ATG2B LC3-II	[54, 103]
miR-124	Osteosarcoma	↓	Camptothecin Etoposide Doxorubicin	DNA repair	ATMIN PARP1	[55]
miR-708	Ewing's sarcoma	↓	Doxorubicin Etoposide	DNA repair	EYA3	[56]
miR-17	Synovial sarcoma	↑	Doxorubicin	Cell cycle	p21	[57]
miR-140	Osteosarcoma	↓	Methotrexate	Cell cycle	HDAC4	[58]
miRNA-215	Osteosarcoma	↑	Methotrexate	Cell cycle	DTL	[59]
miR-301a	Osteosarcoma	↑	Doxorubicin	Evasion of apoptosis	AMPKα1	[63]
miR-382	Osteosarcoma	↓	Doxorubicin Cisplatin Methotrexate	Evasion of apoptosis	HIPK3	[64]
Let-7d	Osteosarcoma	↑	Doxorubicin Cisplatin Paclitaxel Etoposide	Evasion of apoptosis Cancer stem cells	Bcl-2 Caspase-3 Oct3/4 Sox2 Nanog Lin28B HMGA2	[65]
miR-138	Osteosarcoma	↓	Cisplatin	Evasion of apoptosis	EZH2	[66]
miR-21	Osteosarcoma	↑	Cisplatin	Evasion of apoptosis	Bcl-2	[67, 68]
miR-34a	Ewing's sarcoma	↓	Doxorubicin Vincristine	Evasion of apoptosis	Not reported	[69]
miR-125b	Ewing's sarcoma	↑	Doxorubicin Etoposide Vincristine	Evasion of apoptosis	P53 Bak	[74]
miRNA-193a-5p	Osteosarcoma Ewing's sarcoma	↑	Cisplatin	Evasion of apoptosis Activation of cell-survival pathways	TAp73β Wnt/β-catenin pathway	[75, 90]
miR-202	Osteosarcoma	↑	Doxorubicin	Activation of cell-survival pathways	PDCD4	[76]
miR-221	Osteosarcoma	↑	Cisplatin	Activation of cell-survival pathways	PTEN	[82]
miR-217	Osteosarcoma	↓	Cisplatin	Activation of cell-survival pathways	KRAS	[86]
miR-100	Chondrosarcoma	↓	Cisplatin	Activation of cell-survival pathways	mTOR	[87]
miR-497	Osteosarcoma	↓	Cisplatin	Activation of cell-survival pathways	VEGFA	[89]
miR-92a miR-99b miR-422a	Osteosarcoma	↑	Ifosfamide		Wnt/β-catenin pathway	[90]
miR-132	Osteosarcoma	↓	Ifosfamide	Activation of cell-survival pathways	Wnt/β-catenin pathway	[90]
miR-33a	Osteosarcoma	↑	Cisplatin	Activation of cell-survival pathways	TWIST	[91]
miR-34c	Osteosarcoma	↓	Doxorubicin Cisplatin Methotrexate	Activation of cell-survival pathways	North1 LEF1	[93]
miR-146b-5p	Osteosarcoma	↑	Doxorubicin Cisplatin Methotrexate	Activation of cell-survival pathways	ZNRF3	[97]
miR-199a-3p	Osteosarcoma	↓	Doxorubicin	Cancer stem cells	CD44	[102]
miR-29b-1	Osteosarcoma	↓	Doxorubicin Cisplatin Etoposide	Cancer stem cells	Oct3/4 Sox2 Nanog CD133 N-Myc CCND2	[105]
miR-199-5p	Osteosarcoma	↓	Cisplatin	Autophagy	LC3-II Beclin-1	[114]
miR-30a	Osteosarcoma	↓	Doxorubicin	Autophagy	Beclin-1	[115]
miR-22	Osteosarcoma	↑	Doxorubicin Cisplatin	Autophagy	HMGB1	[117, 118]
miR-101	Osteosarcoma	↓	Doxorubicin	Autophagy	LC3 AVOs	[119]
miR-488	Osteosarcoma	↑	Doxorubicin	Evasion of apoptosis	Bim	[145]
miR-184	Osteosarcoma	↑	Doxorubicin	Evasion of apoptosis	Bcl-2	[146]
miR-23b	Chondrosarcoma	↓	Cisplatin	Evasion of apoptosis	Src-Akt pathway	[149]
miR-141	Neuroblastoma	↓	Cisplatin	Not reported	FUS gene	[150]

### miRs are associated with changes to drug targets in drug resistant sarcoma

**Figure 1:**
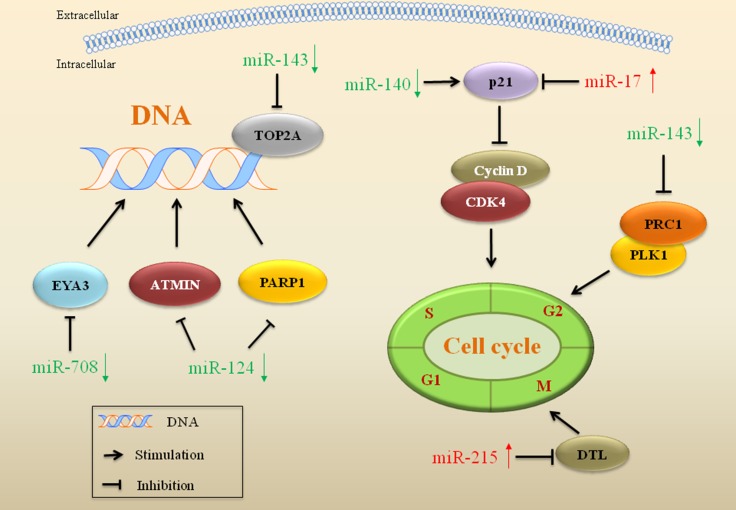
miRs involved in drug target alterations, DNA repair and cell cycle in drug resistant sarcoma miRs that regulate gene expression of crucial proteins in drug targets, DNA repair and cell cycle are implicated in drug resistance in sarcoma. Red arrows indicate upregulation of miRs and green arrows indicate downregulation.

Quantitative or qualitative alterations to drug targets can be a factor contributing to drug resistance. miRs that inhibit DNA topoisomerase 2A (Top2A), such as miR-143, are implicated in drug resistance in dedifferentiated liposarcoma (Figure 1). The miR expression profile showed a decreased miR-143 in dedifferentiated liposarcoma tissues. Restored miR-143 decreased the expression of Top2A, inhibited proliferation and induced apoptosis in dedifferentiated liposarcoma cells. Top2A encoded by the Top2A gene is a nuclear enzyme. It catalyzes the transient breaking and rejoining of two strands of duplex DNA, thus altering the topology of DNA for transcription and replication [52]. The gene encoding Top2A functions as the target for several chemotherapeutic agents used for sarcoma treatment, such as doxorubicin (DOX) and etoposide [53]. Amplification or mutations in this gene have been associated with the development of drug resistance. In dedifferentiated liposarcoma, reduced expression of miR-143 increased nuclear expression of Top2A and cell proliferation, and this led to the dedifferentiated liposarcoma cells acquiring resistance to Top2A-targeted drugs [54].

### miRs influence DNA repair in drug resistant sarcoma

miRs that have an impact on DNA repair, such as miR-124 and miR-708, are implicated in resistance to drugs aimed at damage tumor cell DNA (Figure 1). The damaged DNA leads to genetic instability and in turn activates apoptosis, when DNA repair proves inefficient. Overexpression of miR-124 reduced poly ADP-ribose polymerase 1 (PARP1) and ataxia telangiectasia mutated interactor (ATMIN) proteins expression in osteosarcoma cells, which reduced DNA repair capacity and increased sensitivity to DNA-damaging drugs, such as DOX [55]. Overexpression of miR-124 induced the DNA repair defect through binding to the 3′-UTR of the mRNAs of PARP1 and ATMIN, thus reversed drug resistance in osteosarcoma [55]. Decreased expression of another miR, miR-708, induced EYA3 expression and led to drug resistance in Ewing's sarcoma. EYA3 is a highly expressed transcriptional cofactor that enhances DNA repair in response to DNA damage. Restored miR-708 directly targeted EYA3 and reduced its expression, thus sensitized Ewing's sarcoma cells to the DNA-damaging drugs etoposide and DOX. This confirmed that miR-708 could be a promising target for sensitization to DNA-damaging drugs applied in the treatment of Ewing's sarcoma [56].

### miRs regulate cell cycle-associated genes in drug resistant sarcoma

miRs associated with drug resistance have been shown to regulate genes related to cell cycle in sarcoma, include miR-17, miR-140, miR-215 and miR-143 (Figure 1). Overexpression of miR-17 suppressed DOX-evoked higher expression of p21, a tumor suppressor, and conferred the drug resistance in synovial sarcoma. Treatment of the synovial sarcoma cells with anti-miR-17 rescued expression of p21, which may be a therapeutic target to reverse drug resistance in sarcoma [57]. miR-140 is involved in chemosensitivity with reduced cell proliferation via G1 and G2 phase arrest mediated in part, by the suppression of histone deacetylase 4 (HDAC4) expression. HDAC4 is related to chemoresistance via repression of p21 and regulating cell-cycle progression and differentiation. Transfection with miR-140 reversed methotrexate-resistance through induction of the p21 gene accompanied by G1 and G2 phase arrest only in cell lines containing wild type of p53 [58]. Another miR, miR-215, inhibited expression of denticleless protein homolog (DTL) and led to an increased resistance to methotrexate and Tomudex in osteosarcoma. DTL is a cell cycle G2/M checkpoint regulatory protein. Inhibition of DTL induced G2-arrest and decreased cell proliferation. miR-215 inhibited expression of the DTL gene accompanied by upregulation of p21 resulting in G2-arrest, and thus drug resistance emerged in osteosarcoma [59]. Protein Regulator of cytokinesis 1 (PRC1) is a protein that is highly expressed during S and G2/M phases of the cell cycle [60]. Polo-like kinase 1 (PLK1) or serine/threonine-protein kinase 13 (STPK13), is an early trigger enzyme for G2/M transition [61]. miR-143 was downregulated in dedifferentiated liposarcoma. Restoring miR-143 expression inhibited cytokinesis in dedifferentiated liposarcoma cells, with decreased expression of PRC1 and PLK1. Treatment with a PLK1 inhibitor potently induced G2-M cell cycle arrest and apoptosis in liposarcoma cells, indicating that miR-143 could be a therapeutic target for the reversal of drug resistance [54].

### miRs affect evasion of apoptosis in drug resistant sarcoma

**Figure 2:**
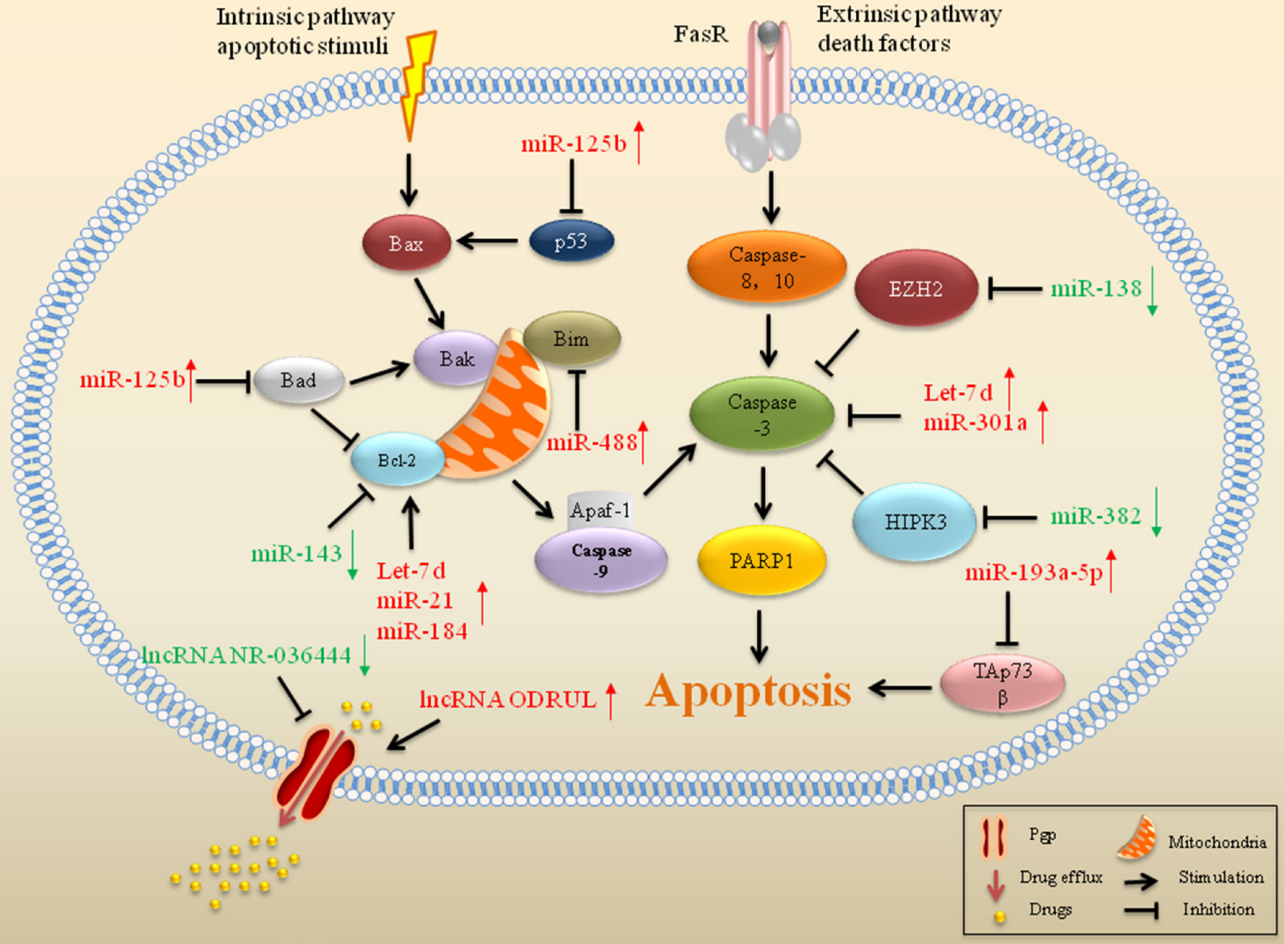
ncRNAs associated with apoptosis and drug efflux in drug resistant sarcoma miRs and lncRNAs that interfere with gene expression of significant proteins in apoptosis and drug efflux have an impact on drug resistance in sarcoma. Red arrows indicate upregulation of miRs and green arrows indicate downregulation.

Dysfunctions of miRs, including miR-301a, miR-382, Led-7, miR-21, miR-34a and miR-125b, are associated with defective apoptosis-induced drug resistance in sarcoma (Figure 2) [62]. DOX treatment promoted the cleavage of caspase-3, which was blocked by miR-301a in osteosarcoma, suggesting that miR-301a enhanced the resistance of osteosarcoma cells to DOX by inhibiting apoptosis [63]. miR-382, increased cleavage of caspase-3 and enhanced drug-induced cell apoptosis in osteosarcoma. Downregulation of miR-382 was associated with a poor chemoresponse to DOX, cisplatin (CDDP), and methotrexate in osteosarcoma [64]. High expression of Let-7d also decreased caspase-3 cleavage induced by chemotherapeutic agents, in combination with reduced apoptosis and drug resistance in osteosarcoma [65]. In contrast, increased miR-138 expression enhanced caspase-3 activation and CCDP-induced apoptosis by targeting EZH2, resulting in reinforcing chemosensitivity to CCDP in osteosarcoma [66].

miR-21 is implicated in the modulation of drug resistance in sarcoma and serum miR-21 can serve as a potential biomarker for prediction of chemotherapeutic sensitivity in patients with osteosarcoma [67]. Transfection with miR-21 mimics depleted the sensitivity of osteosarcoma cells to CDDP, while miR-21 suppression led to enhanced CDDP cytotoxicity. Changes in chemoresponse induced by miR-21 upregulation were ameliorated by downregulation of apoptotic inhibitor Bcl-2 [68]. In addition to miR-21, miR Let-7d also reduced cell sensitivity to DOX, CDDP, etoposide and paclitaxel, consistent with increased expression of Bcl-2 in osteosarcoma [65].

miR-34a was associated with good prognosis in Ewing's sarcoma, and reinforced that miR-34a could sensitize Ewing's sarcoma cells to DOX and vincristine [69]. miR-34a is a component of the p53 tumor suppressor network [70]. Loss of miR-34a is mainly caused by inactivating mutations of p53, genetic alterations of the region Chr1p36 (which contains the coding region for miR-34a), or epigenetic changes [71–73]. miR-125b could induce drug resistance through evasion of p53-dependent apoptosis in DOX-resistant Ewing's sarcoma cells. Knockdown of miR-125b enhanced the sensitivity of Ewing's sarcoma cells to DOX, which was associated with an increased level of p53. In addition to DOX, overexpression of miR-125b also resulted in enhanced resistance to etoposide and vincristine in Ewing's sarcoma [74]. Other members of the p53 tumor suppressor network also influence drug-induced apoptosis in sarcoma. High levels of miR-193a-5p blocked CDDP-induced apoptosis via inhibition of TAp73β, a p53-family protein. The role of the miR-193a-5p/TAp73β axis in drug resistance was confirmed in sarcomas, including in osteosarcoma and Ewing's sarcoma [75].

### miRs interfere with cell-survival pathways in drug resistant sarcoma

**Figure 3:**
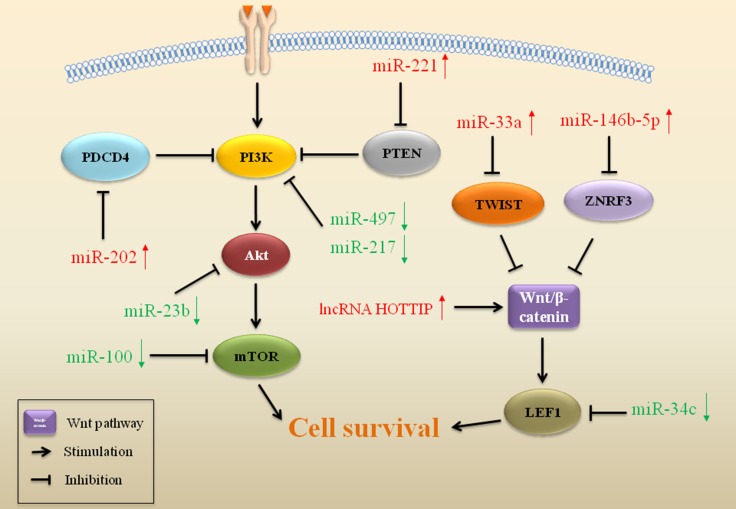
ncRNAs affect cell-survival pathways in drug resistant sarcoma ncRNAs that regulate gene expression of important regulators in cell-survival pathways play a role in drug resistance in sarcoma. Red arrows indicate upregulation and green arrows indicate downregulation of miRs.

Expressions of miRs interfere with cell growth and survival mediated by the PI3K/Akt pathway, which plays a role in drug resistance in sarcoma (Figure 3). To identify PI3K/Akt-related miRs, a group of miRs was shown to have elevated expression upon treatment of drugs, such as miR-202 and miR-221, while other miRs were downregulated, including miR-217, miR-100 and miR-497. Overexpression of miR-202 promoted drug resistance by targeting programmed cell death 4 (PDCD4) in osteosarcoma [76]. PDCD4 is a tumor suppressor that inhibits the PI3K/Akt pathway and enhances apoptosis [77]. Activation of the PI3K/Akt pathway leads to drug resistance, while suppression of PI3K/Akt signaling in turn restores drug sensitivity in cancers, including in sarcoma [78–81]. High levels of miR-221 in osteosarcoma also induced resistance to CDDP and increased cell survival by suppressing PTEN expression [82]. miR-221 directly targeted the 3′-UTR of PTEN and regulated CDDP resistance through the PI3K/Akt pathway with loss of PTEN [82]. As a tumor suppressor, miR-217 increases sensitivity to CDDP through inhibiting the PI3K/Akt signaling pathway in various cancers [83–85]. miR-217 enhanced sensitivity to CDDP via targeting KRAS and inhibiting the PI3K/Akt pathway in osteosarcoma [86]. In chondrosarcoma, reduced expression of miR-100 was associated with resistance to CDDP with activation of the Akt-mTOR pathway. Upregulated expression of miR-100 sensitized chondrosarcoma cells to CDDP by inhibiting the Akt/mTOR pathway [87]. Loss of miR-497 induced drug resistance through targeting vascular endothelial growth factor A (VEGFA) in osteosarcoma. VEGFA is a member of the vascular endothelial growth factor (VEGF) family, which is a positive modulator of the PI3K/Akt pathway [88]. With depletive VEGFA, decreased miR-497 induced Akt activation, resulting in resistance to CDDP. In contrast, increased miR-497 inhibited cell survival and promoted sensitivity to CDDP with inhibition of the PI3K/Akt pathway [89].

The Wnt pathway is another cell-survival signaling pathway associated with drug resistance in sarcoma (Figure 3). miR profiles identified five discrete miRs (miR-92a, miR-99b, miR-132, miR-193a-5p and miR-422a) in osteosarcoma, which have an impact on drug response through targeting the Wnt, TGFβ and MAP kinase pathways [90]. Overexpression of miR-33a also resulted in drug resistance in osteosarcoma, which was mediated by downregulation of TWIST [91]. TWIST is a negative regulator of CDDP resistance by suppressing the Wnt/β-catenin pathway in osteosarcoma [92]. Low expression of miR-34c contributed to drug resistance development in osteosarcoma [93]. miR-34c may target and inhibit Notch1 and LEF1 expression. Notch1 and LEF1 activated the Notch and the Wnt signaling pathway, promoting drug resistance in cancers, including in osteosarcoma [94–96]. Overexpression of miR-146b-5p promoted resistance to DOX, CDDP and methotrexate by promoting the Wnt/β-catenin pathway in osteosarcoma. Zinc and ring finger 3 (ZNRF3) is a direct target of miR-146b-5p. Overexpression of miR-146b-5p downregulated ZNRF3, thus activate Wnt/β-catenin pathway and induced drug resistance in osteosarcoma [97].

### miRs are involved in CSC-related pathways in drug resistant sarcoma

**Figure 4:**
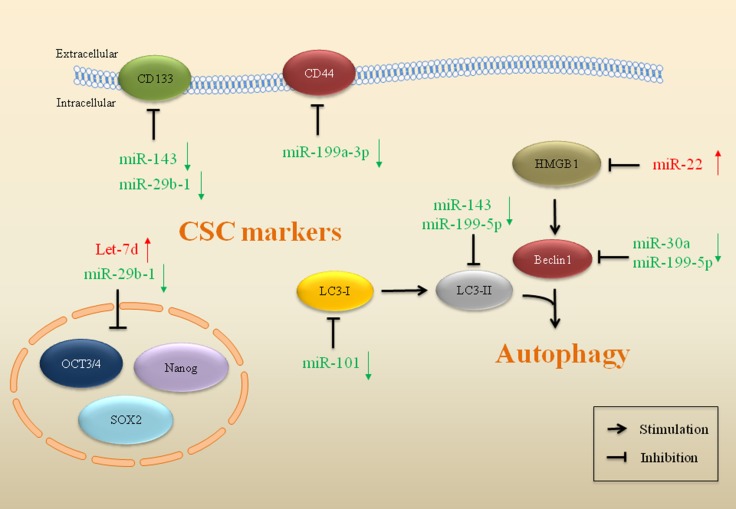
miRs impact on CSCs and autophagy in drug resistant sarcoma miRs that mediate gene expression of markers and regulators in CSCs and autophagy are implicated in drug resistance in sarcoma. Red arrows indicate upregulation of miRs and green arrows indicate downregulation.

The CSC hypothesis postulates that within a tumor, a minor subpopulation of cells possess the capacity to self-renew and drive the heterogeneous lineages of cancer cells that account for the initiation, proliferation, recurrence, metastasis and therapeutic resistance in cancer [98]. CSCs may be involved in resistance to drugs and toxins through the expression of drug efflux transporters, activation of DNA repair, and evasion of apoptosis [99]. For example, CSCs express more anti-apoptotic proteins, including Bcl-2, FLIP, IAP-1, IAP-2 and survivin than normal tumor cells to resist apoptosis in sarcoma [100]. Even though the molecular pathways modulating CSCs remain unclear, growing evidence highlights the roles of miRs in the regulation of drug resistance related to CSCs (Figure 4).

Cluster of differentiation 44 (CD44) is a cell surface marker commonly used to identify and enrich CSCs [101]. Higher expression of this cell-surface glycoprotein was associated with response to chemotherapy in osteosarcoma. miR-199a-3p is one of the most dramatically decreased miRs in osteosarcoma. Transfection of miR-199a-3p enhanced drug sensitivity, consistent with downregulation of CD44 in osteosarcoma. The 3′-UTR of CD44 mRNA was the direct target of miR-199a-3p [102]. In chemoresistant osteosarcoma cell lines, it was also observed that loss of miR-143 expression was accompanied by a high level of ALDH1^+^CD133^+^ cells. Forced miR-143 expression could reverse drug resistance in DOX-resistant osteosarcoma CSCs through activation of apoptosis [103]. CSCs in sarcoma can also induce evasion of apoptosis through p53 and Rb pathways to resist chemotherapeutic drugs [104]. The AB-OS cell line is a CSC cell line selected from human osteosarcoma MG63 cells. Overexpression of miR-29b-1 reduced the expression of stem cell markers (Oct3/4, Sox2, Nanog, CD133, and N-Myc) and anti-apoptotic markers (Bcl-2 and IAP-2) in 3AB-OS CSCs, suggesting that miR-29b-1 can inhibit stemness properties of 3AB-OS CSCs and sensitize 3AB-OS cells to chemotherapy drugs (DOX, CDDP and etoposide) in osteosarcoma [105]. On the contrary, upregulation of Let-7d reduced cell sensitivity to apoptosis and induced drug resistance in 3AB-OS cells, in combination with decreased caspase-3 and increased Bcl-2 in osteosarcoma [65].

### miRs impact autophagy in drug resistant sarcoma

Recent findings have suggested that dysregulated miRs are closely connected to autophagy modulation and cancer drug resistance (Figure 4). Autophagy is an evolutionary conserved catabolic process involving in the degradation of proteins and organelles, cellular remodeling and survival, promoting the controlled degradation of cytoplasmic material both at steady state and during nutrient deprivation [106]. Tumor cell autophagy is activated during chemotherapy and contributes to chemotherapy resistance [107, 108]. Thus, inhibition of autophagy can re-sensitize resistant cancer cells and enhance cytotoxicity of chemotherapeutic agents [109, 110]. Certain miRs have been revealed as potent inhibitors of drug resistance-related autophagy [111], indicating their potential role in cancer therapy [112, 113].

It has been shown that miR143 and miR-199-5p suppress autophagy and reverse drug resistance in osteosarcoma (Figure 4). Upregulation of miR-143 was correlated with the sensitization of osteosarcoma cells to DOX, which is accompanied by downregulation of LC3-II and Bcl-2 [103]. CDDP treatment upregulated protein levels of LC3-II and Beclin1 in osteosarcoma cells, indicating that autophagy was activated. Restoration of miR-199a-5p inhibited CDDP-induced autophagy, indicating that miR-199a-5p promotes the cytotoxicity of CDDP in osteosarcoma through inhibition of autophagy [114]. Reduced miR-30a expression activated Beclin-1-dependent autophagy, which influenced drug resistance in osteosarcoma cells to DOX. miR-30a directly binds to the 3′-UTR of Beclin-1 gene, resulting in the suppression of Beclin-1 mediated autophagy. Elevated miR-30a expression promoted drug-induced apoptosis and inhibited autophagy activity in osteosarcoma [115, 116]. The expression of miR-22 was induced by chemotherapeutic drugs in osteosarcoma. High-mobility group box 1 (HMGB1) is a direct target of miR-22, which could facilitate autophagy and promote drug resistance. miR-22 blocked the HMGB1-mediated autophagy and reversed drug resistance in osteosarcoma [117, 118]. Autophagy-characteristic acidic vesicular organelles (AVOs) are characteristic in autophagy. miR-101 could also reduce the DOX-induced AVOs and decrease autophagy-related proteins in osteosarcoma. Upregulated miR-101 enhanced chemosensitivity to DOX through inhibiting autophagy, thus reversing autophagy-mediated drug resistance in osteosarcoma [119].

## ROLE OF LNCRNAS IN DRUG RESISTANT SARCOMA

LncRNAs are transcriptional polyadenylated RNAs that were first reported in 1990 [120]. They consist of exons and introns in structure, but lack valid ORFs [22]. LncRNAs regulate gene expression at epigenetic, transcriptional and post-transcriptional levels, through epigenetic regulation, splicing, imprinting, transcriptional regulation and subcellular transport [121–124]. They function in both *cis*, to act on the same chromosome, and *trans*, to affect different chromosomes [125, 126]. LncRNAs play a role in a number of biological processes, including chromatin modification, telomere biology and subcellular structural organization [127]. Current estimates of unique lncRNAs present in humans that range from 7000–23,000, with a growing cohort being validated as having a role in human disease processes, including in sarcoma [128, 129]. The misregulated lncRNAs can function as decoys, scaffolds, signals or guides for specific regulatory modules, resulting in a gene expression profile in favor of cancer drug resistance development (Table 3) [24, 130].

**Table 3:** Summary of lncRNAs involved in drug resistant sarcoma

lncRNAs	Sarcomas	Alteration	Drugs	Resistance mechanisms	Major targets	References
ENST00000563280 (ODRUL)	Osteosarcoma	↑	Doxorubicin	Evasion of apoptosis	ABCB1	[131, 137]
NR-036444	Osteosarcoma	↓	Doxorubicin	Evasion of apoptosis	ABCB1	[131]
HOTTIP	Osteosarcoma	↑	Cisplatin	Activation of cell-survival pathways	Wnt/β-catenin pathways	[139]

### lncRNAs are associated with drug efflux and accumulation in drug resistant sarcoma

A human lncRNA-mRNA combined microarray revealed that 3,465 lncRNAs (1,761 up and 1,704 down) were aberrantly expressed in DOX-resistant osteosarcoma cells. This lncRNA-mRNA co-expression network identified that upregulated expression of lncRNA EST00000563280 and downregulated expression of lncRNA NR-036444 could stimulate the expression of Pgp to transport the drugs out of the cell, leading to drug resistance to DOX in osteosarcoma (Figure 2) [131]. Pgp, also known as MDR1, is a 170 kDa cell membrane glycoprotein encoded by the ATP-binding cassette subfamily B member 1 (ABCB1) gene. ABCB1 is a member of the ubiquitous ATP-binding cassette (ABC) superfamily, which contains 49 members divided into seven subclasses ranging from ABCA to ABCG [132]. As an ATP-dependent drug transporter, Pgp can extrude cytostatic agents against a drug concentration gradient at the expense of ATP hydrolysis. The action of Pgp triggers drug efflux and promotes resistance to drugs in cancer [133, 134]. In sarcoma, the acute induced expression of Pgp has been demonstrated as a function of time in response to *in vivo* exposure to chemotherapy [135, 136]. The novel lncRNA EST00000563280, termed osteosarcoma DOX-resistance related upregulated lncRNA (ODRUL), was the most upregulated lncRNA in DOX-resistant osteosarcoma cells. ODRUL plays a role in the emergence of resistance to DOX by inducing Pgp expression in osteosarcoma [137]. This indicates that lncRNAs may be a novel target for reversing drug resistance in sarcoma.

### lncRNAs regulate cell-survival pathways in drug resistant sarcoma

In addition to promoting cancer cell survival, activation of Wnt/β-catenin pathway can also induce drug resistance through affecting cell cycle in osteosarcoma (Figure 3) [138]. HOTTIP is another lncRNA upregulated in osteosarcoma. Overexpression of HOTTIP promoted cellular resistance to CDDP, accompanied by an activation of Wnt/β-catenin pathway. Decreasing HOTTIP induced cell cycle arrest in G1 phase through inhibiting the Wnt/β-catenin pathway, thus reversing drug resistance in osteosarcoma [139].

### The therapeutic potential of ncRNAs in drug resistant sarcoma

Identification of chemotherapy-resistant tumors at diagnosis is a first priority for treating sarcoma, where more individualized, effective, and less toxic treatments are highly desirable. ncRNA therapiesappear to be a novel field in which ncRNA activity is the major target of the intervention [140].

miRs are believed to be relatively safe and more effective in cancer treatment in early preclinical studies [141]. Recent evidence has confirmed that the selective modulation of miR activity could improve responses to chemotherapy. The rationale for developing miR therapies is based on the premise that aberrant expression of miRs play key roles in the development of resistance to chemotherapy and that correcting these miR deficiencies by either antagonizing or restoring miR function may provide a therapeutic benefit [142].

Overexpressions of some miRs are involved in drug-resistant human sarcoma. Inhibition of these miRs may increase chemoresponse to antitumor therapy. For example, miR-21 promotes drug resistance in sarcoma, and inhibition of miR-21 may function to reverse drug resistance in osteosarcoma cells [68]. Further understanding of miR-21-mediated signaling pathways will help to promote the clinical use of miR-21 in cancer treatment [143]. DOX is a widely used chemotherapy drug, but it has a narrow therapeutic window with toxic side-effects. The antitumor activity of DOX is achieved by induction of cell apoptosis through interacting with DNA [144]. The miR-488 is overexpressed in osteosarcoma and is induced by hypoxia through binding to the hypoxia response element. Overexpression of miR-488 decreased the sensitivity to DOX via direct targeting of the mediator of apoptosis Bim. Transfection of the miR-488 inhibitor resulted in an increase in apoptosis and enhanced the sensitivity of osteosarcoma cells to DOX treatment [145]. Overexpression of Let-7d also reduced osteosarcoma cell sensitivity to apoptosis induced by chemotherapy agents DOX, CDDP, etoposide and paclitaxel, concomitant with a decrease in caspase-3 and increase in BCL2 expression [65]. miR-184 also contributes to chemoresistance in osteosarcoma. The expression of miR-184 was upregulated in DOX-treated osteosarcoma cells. BCL2L1 served as the direct target gene of miR-184. Overexpression of miR-184 reduced DOX-induced cell apoptosis and led to poor response to drug therapy by targeting BCL2L1 [146]. These observations indicate that miR-488, Let-7d and miR-184 may serve as predictors of response to chemotherapy and as potential therapeutic targets for the treatment of drug resistant osteosarcoma.

Some miRs are downregulated and related to drug resistance in sarcoma. Exogenous upregulation of these may promote sensitivity to chemotherapy. In Ewing's sarcoma, miRNA expression was investigated in patients with different clinical outcomes. Microarray analysis identified a signature of miR-34a as a prognostic indicator for Ewing's sarcoma [147]. Functional analysis indicated that miR-34a increased tumor sensitivity to the effects of DOX and vincristine in Ewing's sarcoma cells, and that restoration of miR-34a activity may be useful to decrease malignancy. The result suggested a potential role of miR-34a in the treatment of sarcoma and may spare excessive long-term toxicity to Ewing's sarcoma patients [69]. Chondrosarcoma is the second most common type of primary bone malignancy in the United States. The expression of miR-125b was decreased in both parental and DOX-resistant chondrosarcoma cells. Overexpression of miR-125 enhanced the sensitivity of both parental and DOX-resistant cells to DOX by inhibiting glycolysis through directly targeting the oncogene, ErbB2 [148]. CDDP is another widely used antitumor agent via effective DNA-damaging. However, chondrosarcoma is resistant to conventional chemotherapies, including CDDP. Compared with the parental cells, miR-23b was significantly downregulated in CDDP-resistant cells. Src kinase is a direct target of miR-23b in chondrosarcoma cells. The chemo-sensitization effect induced by miR-23b-mediated suppression of the Src-Akt pathway suggests that miR-23b could be a potential therapeutic target to reverse the acquirement of resistance to CDDP in chondrosarcoma [149]. These findings provide a novel therapy to overcoming chemoresistance in human chondrosarcoma cells. In neuroblastoma, miR-141 upregulation promoted chemosensitivity to CDDP, which was associated with the FUS gene, which may also help in the development of therapeutic strategies for the treatments of patients [150].

Moreover, lncRNAs have become a new frontier for drug development. The structural and functional novelty of lncRNAs offers promise as anticancer therapeutics that may avoid the emergence of drug resistance commonly seen with the currently used agents. For example, HOTAIR is a novel factor involved in drug resistance and metastasis in various types of solid tumors. Overexpression of HOTAIR was involved in drug resistance in primary sarcoma and decreased levels of HOTAIR were associated with good chemoresponse, suggesting that clinical application of HOTAIR may be a potential predictor of treatment efficacy [151].

Although there are still several obstacles to overcome before clinical testing of miR therapeutics, such as delivery and chemical modification of ncRNA modulators, it can be expected that ncRNAs and ncRNA-targeting oligonucleotides may become promising tools in the fight against drug resistant sarcoma in the near future [152].

## CONCLUSIONS AND FUTURE PERSPECTIVES

The current rapid expansion of research on ncRNAs evidently shows that ncRNAs have many implications in drug resistant sarcoma. This review brings together information on the roles of ncRNAs in drug resistant sarcomas, including miRs and lncRNAs. Although the exact mechanisms of ncRNAs in drug resistance in sarcoma are still unclear, these studies highlight the increasing interest and development of knowledge of ncRNAs in drug resistant sarcoma.

Although recent findings present a positive trend in development, the research progress of ncRNAs in drug resistant sarcoma is still in its infancy. There is a tough route from discovering the function of ncRNAs to its clinical use. Despite extensive investigation and distinct progress in this field, there are still many challenges. Most of the current studies focus on the role of ncRNA in chemoresistance with a single agent, such as either DOX or CDDP. However, sarcoma patients commonly receive combined chemotherapy in the clinic. Thus, it is necessary to evaluate the role of ncRNA in the regulation of multidrug resistance. With regard to ncRNA-based therapeutic agent, the field will benefit from the development of RNA delivery technology and chemical modifications of ncRNAs. Novel RNA delivery technology may protect ncRNA from degradation and enable ncRNA to reach tumors efficiently and specifically. The chemical modifications of ncRNAs can enhance the stability of ncRNAs and reduce toxicity, minimizing side effects. Furthermore, discovery of more miR targets will be useful to better identify new and more effective drug targets [153].

A rising strategy to solve gene specificity limitations is the technology of genome editing by clustered regulatory interspaced short palindromic repeats-associated endonuclease 9 (CRISPR-Cas9), which is a rapid and efficient way to generate total or partial downregulation of specific genes, including ncRNAs, by the targeted interruption of the promoter and the chosen sequence through insertion of polyadenylation signals. Furthermore, CRISPR-Cas9 can be applied to achieve ncRNA overexpression from its endogenous locus by inserting a strong promoter upstream of the ncRNA sequence or by targeting transcriptional activator complexes to the promoter [154, 155]. In the future, after gaining insight into the ncRNAs involved in drug resistance mechanisms in sarcoma, ncRNA-based approaches may provide important advances in overcoming drug resistance and improving chemotherapy responses of sarcoma patients.
